# The effect of pre-operative high doses of methylprednisolone on pain management and convalescence after total hip replacement in elderly: a double-blind randomized study

**DOI:** 10.1007/s00264-020-04802-8

**Published:** 2020-09-17

**Authors:** Artur Gądek, Henryk Liszka, Małgorzata Zając

**Affiliations:** 1 Department of Orthopedics and Physiotherapy, Jagiellonian University Collegium Medicum, Krakow, Poland; 2 Department of Orthopedics and Traumatology, University Hospital in Krakow, Krakow, Poland; 3 Department of Anatomy, Jagiellonian University Medical College, Krakow, Poland; 4 Department of Anaesthesiology and Intensive Care, Jagiellonian University Collegium Medicum, Krakow, Poland; 5 Department of Anaesthesiology and Intensive Care, University Hospital in Krakow, Krakow, Poland

**Keywords:** Total hip joint replacement, Methylprednisolone, Opioid dose, Pain intensity, Infection rate

## Abstract

**Purpose:**

The aim of the study was to assess whether administration of a single dose of methylprednisolone in the group patients above 65 years of age will be effective in complex analgesic management after total hip arthroplasty (THA).

**Methods:**

Seventy-seven patients above 65 years old were double-blind randomized into two: the study and controls groups. Pre-operatively, the study group received as a single dose of 125 mg intravenous methylprednisolone, while the others saline solution as placebo. Peri-operatively, all the patients were administered opioid and nonopioid analgesic agents. We measured the levels of inflammatory markers (leukocytosis, C-reactive protein—CRP), pain intensity level (visual analog scale—VAS; numerical rating scale—NRS), the life parameters, and noted complications.

**Results:**

Following administration of methylprednisolone were significantly lower levels of CRP on all the four post-operative days; leukocytosis on the second day; the VAS/NRS score at rest after six, 12, and 18 hours post-operatively, diminished the dose of parenteral opioid preparations (oxycodone hydrochloride), the duration of analgesia by peripheral nerve block was significantly higher as compared with the placebo group (*p* < 0.000001). No infectious complications were noted; there was one patient who developed post-operative delirium.

**Conclusion:**

A single dose of methylprednisolone significantly reduces the level of post-operative pain at rest on the day of THA in the group patients above 65 years of age, decreases the dose of opioid analgesic agents, and significantly decreases the level of inflammatory markers, without infectious processes.

## Introduction

Total hip and knee arthroplasties are associated with considerable pain between the sixth and 24th post-operative hour [1–3]. The surgical incision, as a mechanical factor, affects the nociceptor, initiates the pain process, and triggers numerous inflammatory mediators [1, 4, 5]. The Enhanced Recovery After Surgery (ERAS) with the method multimodal analgesia accelerates the patient’s returning to full activity [4–10] and limits the complications and time of hospitalization [9–14]. Of interest is whether ERAS protocol is effective in the group patients older than 65 years, who more commonly suffer from concomitants diseases and whose comorbidities are more advanced; the physiological functional reserve is decidedly lower due to systemic involution [10–13, 15].

The management aiming at reducing the inflammatory process and pain for chronic osteoarthritis is commonly administered intra-articular glucocorticoid injections, hence the interest, whether administration only once, as a “pre-emptive” analgesia, may effectively limit acute pain, will not the immunosuppressive effect resulting among others from blocking the elements of the native immune system, increase the risk of infectious complications developing? Numerous reports have indicated an effective use of methylprednisolone as an element of multimodal analgesia in orthopaedic surgery [1, 9, 16–21].

## Objective

The objective of the study was the effectiveness of methylprednisolone administration in complex peri-operative analgesic therapy in patients above 65 years of age, subjected total hip arthroplasty (THA). The authors attempted to answer the questions whether a one-time administration of methylprednisolone will reduce the following: (1) the pain level in the post-operative period, the value of visual analog scale (VAS), and numerical rating scale (NRS) at rest at every six hours on the day of surgery; (2) the dose of the parenteral analgesic agents, including opioids; (3) the mobilization of inflammatory process parameters; (4) the occurrence of adverse effects that would delay early rehabilitation on the ERAS protocol; (5) maintain a stable glycemia level.

## Material and methods

The analysis included a group of 92 patients above 65 years of age, subjected to elective unilateral THA, performed in keeping with the ERAS protocol in the period from January 1 to June 30, 2019. Fifteen patients were excluded from the study due to clinical situations that limited the use of a glucocorticoid: 1/diabetes type 1 and 2; 2/rheumatoid arthritis; 3/when C-reactive protein (CRP) levels were above normal values ≥ 5 mg/L; 4/chronic steroid treatment; 5/peptic ulcers within the past 30 days.

The patients were subjected to the standardized spinal anaesthesia procedure, with subsequent fascia iliaca compartment blockade at the operated side, followed by THA from the lateral approach. Prior to anaesthesia induction, the patients received intravenous agents: an infection-preventing (cephazolin 2.0 g), in order to achieve haemostasis (tranexamic acid 1.0 g), and an antiemetic control (ondansetron 8 mg). The fluid intake (crystalloids) was standardized: 12 mL/kg/h in the first hour and 6 mL/kg/h in the following hours of operation, erythrocyte mass was packed, red blood cells were transfused, when blood loss exceeded 600 mL. Pain management was determined on the VAS/NRS tools at rest every six hours; if the pain score was ≥ 4 points, oxycodone hydrochloride at the subcutaneous dose of 0.1 mg/kg of body weight was administered; if the pain score was 2–4 points, paracetamol and metamizole were calculated for 1 kg of body weight. In keeping with the ERAS protocol, on the day of surgery, the patients received fluids and meals if no post-operative nausea and vomiting (PONV) occurred and were rehabilitated.

The study was approved by the institutional review board (nr 1072.6120.11.2020). Following their giving oral and written consent to participate in the investigation, all the patients included in the study and subjected to the analysis were randomized into two groups: the study group and the controls using the double blind method. The study group (M) received methylprednisolone at the intravenous dose of 125 mg as “pre-emptive” analgesia, and the controls (K) saline solution as placebo. The statistical analysis included demographic dates, general condition in the ASA (American Society of Anesthesiology), POSSUM (Physiologic and Operative Severity Score for the enUmeration of Mortality and Morbidity), total dose of analgesic medications administered parenterally calculated for 1 kg of body mass in response to value of VAS/NRS at rest on day zero, time of administration of the first dose, and duration of fascia iliaca compartment blockade. On the day of surgery and on subsequent days, determinations were made for glycemia and inflammatory markers: CRP and leukocytosis levels.

The statistical analysis was performed using the Student *T* test for independent groups employing the Cochran-Cox modification; the resultant statistical significance was *p* < 0.05 employing the PQStat v 1.6.8 software.

## Results

The analyzed groups were comparable as to the number of subjects: M group (methylprednisolone)—39; K group (placebo)—38 patients. There were no inter-group differences in the mean age, post-operative duration of hospitalization, duration of the procedure, intra-operative blood loss, and life parameters on the day of surgery. The groups differed in their peri-operative risk value as measured by the ASA and POSSUM scale (Tables 1 and 2).

**Table 1:** General data describing the analyzed elderly patients (over 65 years old) divided into the study group (M) and the controls (K)

Data	Group (M)	Group (K)	*p* value
*n*	39	38	
Age (years)	72.66 ± 6.92	72.71 ± 5.93	0.97
Mean duration of postoperative hospitalization (days)	4.89 ± 2.64	5.47 ± 2.82	0.35
ASA*	II—76.93%; III—23.07%	II—42.1%; III—57.9%	
Operative MAP^ (mmHg)	93.82 ± 18.97	93.62 ± 16.48	0.988
Operative heart rate (x/min)	69.42 ± 16.65	73.75 ± 17.23	0.192
Post-operative MAP^ (mmHg)	93.04 ± 9.27	94.75 ± 14.67	0.947
Post-operative heart rate (x/min)	71.84 ± 6.73	75.0 ± 11.03	0.238
Post-operative Sp02 (%)	98.4 ± 0.89	98.05 ± 12.93	0.974

*American Society of Anesthesiologists Score

^Mean arterial pressure

**Table 2:** Distribution of comorbidities and anaesthesia types in the analyzed patients above 65 years of age divided into the study group (M) and the controls (K)

Data	Group M		Group K		*p* value
	*n*	%	*N*	%	
*n*	39	100	38	100	
Hypertension arterial	39	100	38	100	
Atrial fibrillation, arrhythmia supraventricular	6		9		
IHD, myocardial infarction in anamnesis	5		8		
COPD grade III	2		5		
TIA in anamnesis	0	0	1		
Circulatory failure chronic	5		9		
Regional anesthesia	39	100	38	100	
Intercompartmental nerve block analgesia	39	100	38	100	
ASA^ III	9	23.07	22	57.9	0.001537
POSSUM* physiological (points)	17.30 ± 3.88		20.00 ± 3.99		0.0036
POSSUM* morbidity (%)	24.57 ± 12.50		33.17 ± 15.49		0.0089
POSSUM* mortality (%)	1.53 ± 1.56		2.15 ± 1.63		0.09

*IHD* ischemic heart disease, *COPD* chronic obstructive pulmonary disease, *TIA* transient ischemic attack

^American Society of Anesthesiology Score,

*Physiological and Operative Severity Score for the enumeration of Morbidity and Mortality

**Fig. 1:**
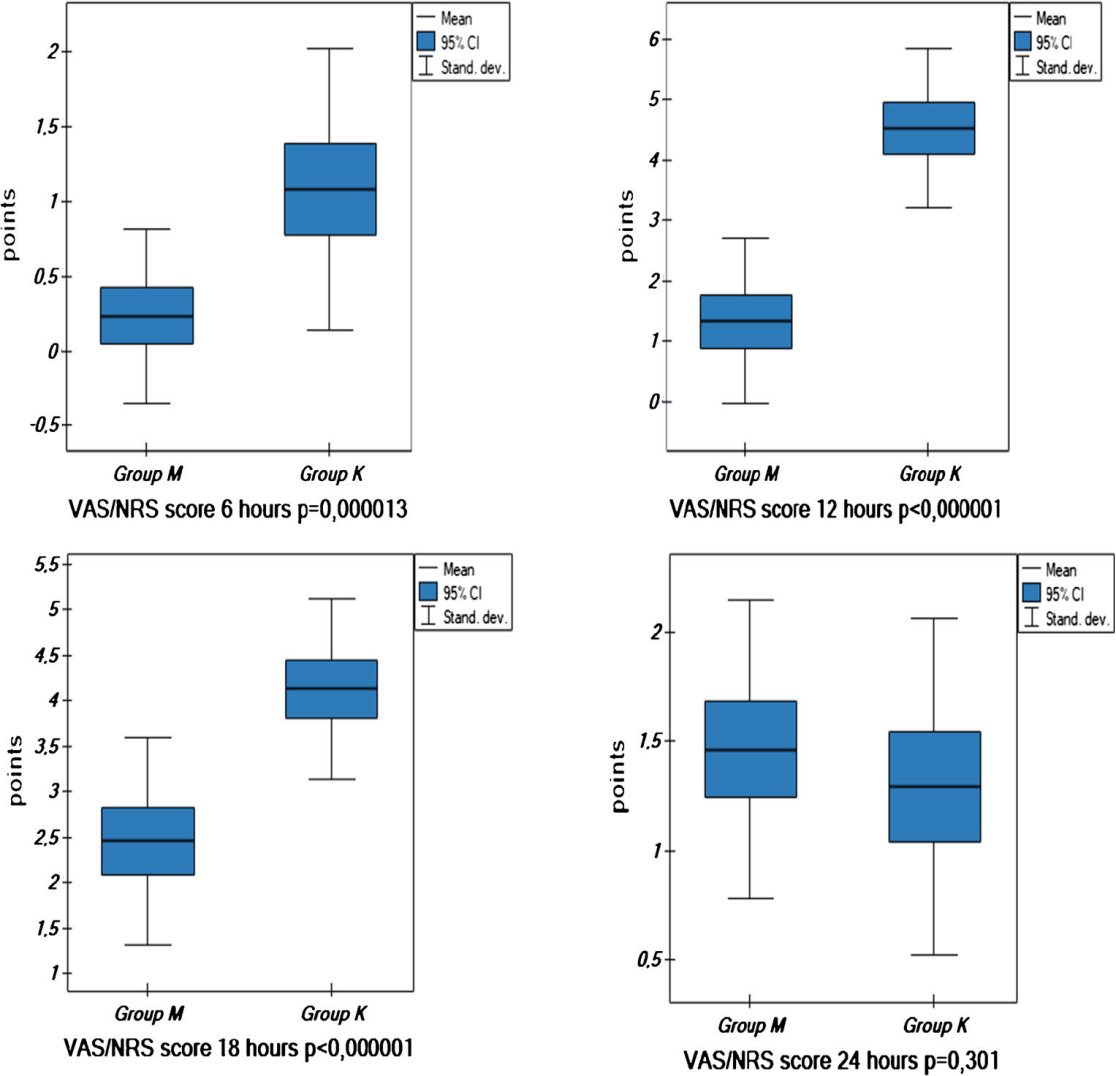
VAS/NRS values at rest presented as scores at 6, 12, 18, and 24 h post-operatively in the analyzed patients above 65 years of age divided into the study group (M) and the controls (K)

**Fig. 2:**
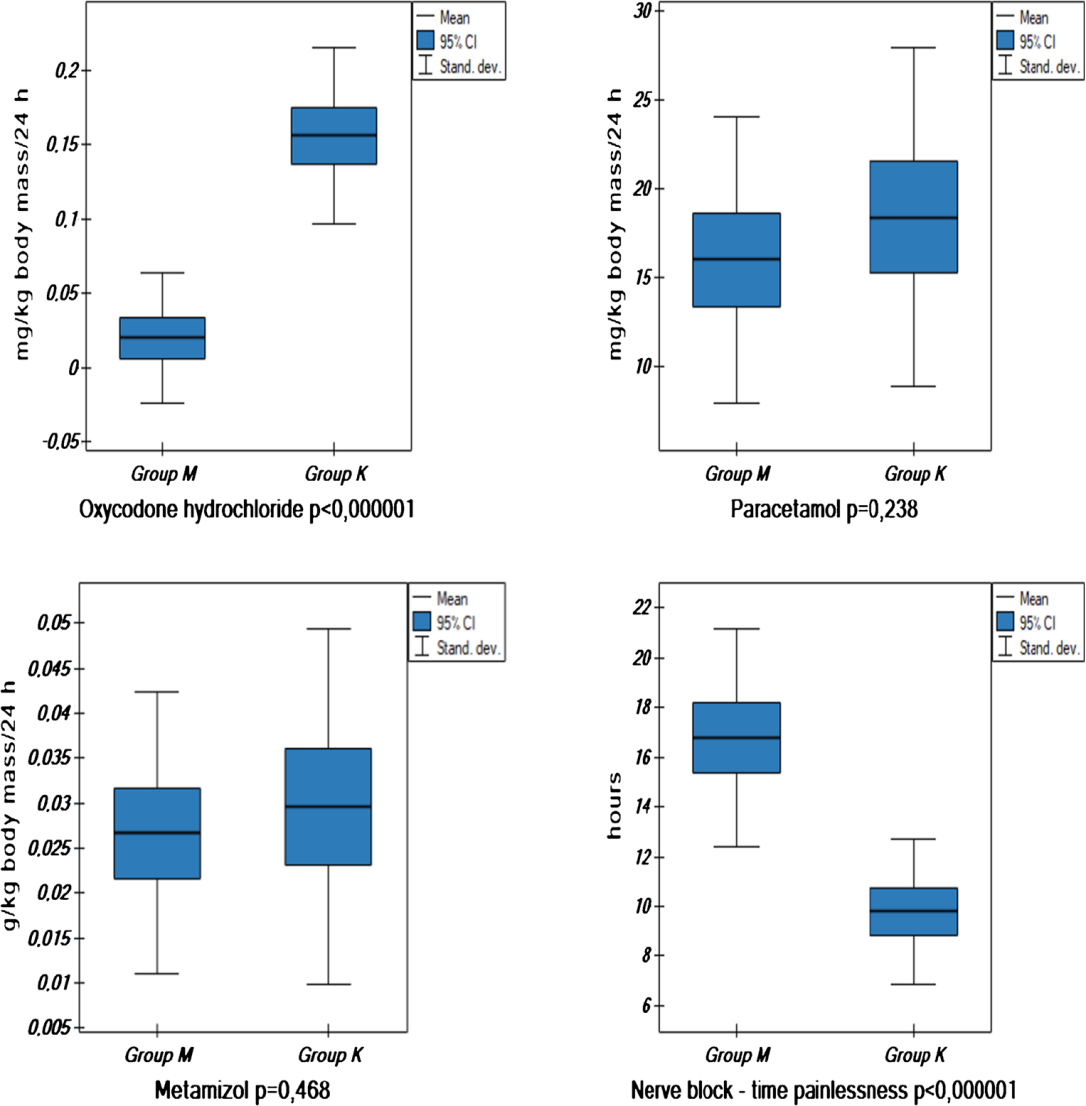
Doses of analgesic medications and duration of post-operative analgesia not requiring analgesics administration on the day of surgery in the analyzed patients above 65 years of age divided into the study group (M) and the controls (K)

In the post-operative period, the analysis included in the two groups: among inflammatory markers the leukocytosis, blood CRP levels on day zero, one, two and three post-operatively, and CRP level in the surgical wound drainage on day 0; glycemia levels on consecutive post-operative days; doses of analgesic medications administered on the day 0; and the duration of peripheral nerve block that was tantamount to the time of administration of the first analgesic agent dose (Table 3, Fig. 1 and Fig. 2).

**Table 3:** Leukocytosis, C-reactive protein (CRP), and glucose levels in the analyzed patients aged above 65 years old divided into the study (M) and control (K) groups

Data	Group (M)	Group (K)	OR	− 95 CI	*p* value
*n*	39	38			
Initial leukocytosis (× 10^3^/μL)	7.58 ± 2.5	7.70 ± 1.91	− 0.12	− 1.04/0.80	0.80
Leukocytosis on day zero (× 10^3^/μL)	12.11 ± 3.25	10.84 ± 2.18	1.27	0.013/2.53	0.0477
Leukocytosis on day 1 (× 10^3^/μL)	12.99 ± 3.64	12.02 ± 2.26	0.97	− 0.40/2.35	0.163
Leukocytosis on day 2 (× 10^3^/μL)	10.39 ± 2.81	11.84 ± 2.51	− 1.45	− 2.67/−0.24	< 0.0193
Leukocytosis on day 3 (× 10^3^/μL)	8.94 ± 2.04	11.59 ± 1.68	− 2.66	− 3.52/− 1.80	< 0.000001
Initial CRP (mg/L)	2.77 ± 1.55	2.97 ± 1.60	− 0.19	− 0.92/0.53	0.5888
CRP on day zero (mg/L)	19.34 ± 23.6	41.91 ± 22.69	− 22.58	− 33.09/− 12.06	0.000056
CRP on day 1 (mg/L)	46.67 ± 25.71	102.19 ± 34.1	− 55.52	− 69.28/− 41.76	< 0.000001
CRP on day 2 (mg/L)	72.61 ± 46.87	144.40 ± 54.39	− 71.79	− 94.88/− 48.70	< 0.000001
CRP on day 3 (mg/L)	73.11 ± 43.96	140.75 ± 56.78	− 67.65	− 90.88/− 44.41	< 0.000001
CRP drain (mg/L)	6.24 ± 8.48	9.73 ± 14.70	− 8.98	− 8.98/2.00	0.20
Initial glucose (mmol/L)	5.60 ± 0.81	5.92 ± 0.74	− 0.32	− 0.68/0.04	0.084
Glucose on day zero (mmol/L)	6.80 ± 0.99	6.85 ± 0.85	− 0.05	− 0.47/0.36	0.796
Glucose on day 1 (mmol/L)	6.27 ± 0.97	6.60 ± 0.78	− 0.33	− 0.73/0.07	0.104
Glucose on day 2 (mmol/L)	5.78 ± 0.81	5.85 ± 0.69	− 0.065	− 0.42/− 0.28	0.706
Glucose on day 3 (mmol/L)	5.92 ± 0.57	6.10 ± 0.56	− 0.18	− 0.44/0.07	0.158

*CRP* C-reactive protein, *OR* odds ratio, *CI* confidence interval

The duration of fascia iliaca compartment blockade in the group M was significantly longer—only 8 patients (20.51%) received oxycodone hydrochloride, in the group K 36 subjects (94.73%) in view ≥ 4 VAS/NRS pain level at rest (Fig. 2).

## Discussion

The ERAS protocol with satisfactory multimodal analgesia is becoming a standard following the major orthopaedic procedures, shortens the post-operative time of hospitalization, and also makes it possible to promptly mobilize and effectively rehabilitate patients on the day of operation. Short hospitalization decreases the risk of infectious complications evoked by hospital bacterial strains [2, 11, 12, 15, 18, 19, 21, 22], as well as thrombotic, circulatory, and respiratory complications. Reports are published that describe replacement of major joints performed as “same day” procedures [9, 10, 13].

In the present material, the mean post-operative hospitalization time was 4.89 days (2–17 days) in the study group and 5.47 days (2–19 days) in the controls (*p* = 0.35, Table 1). Prolonged hospitalization time involved one patient in each group, at the age 91 and 94 years with the difficulties in rehabilitation due to delirium. Significantly shorter time of the post-operative hospitalization following arthroplasty after entering the ERAS procedure presents a meta-analysis by Pamilo et al. (10) from Orthopedic Wards Finland—three  days in the period of 2012–2013 vs. five days 2009–2010; by Memtsoudis et al. from 550 Europe’s and United States’s hospitals—three  days in the period of 2006–2014; and by Farley et al.—4.06 days before 2002 and 2.97 days in years 2012–2014 [10, 15, 23].

Osteoarthritis of the hip joint  has developed over many years, restricts physical activity, and affects patients more in their sixth - seventh decade of life. According to Ghironzi et al., the elderly age is the age when the progressive physiological decrease of the functional reserve occurs, leading to increasing incidence of diseases, mortality, and a decreased reproduction rate. Hence, the group of individuals from 85 years of life is at present more frequently recognized as the elderly age group [24, 25]. After 65 years of age, involution processes become significantly intensified; the 65th year of life marks the beginning of the subject’s so-called elderly patients [12, 13, 24, 25]. The analyzed material represents the patients above 65 years. The initial condition of the patients differed in the two analyzed groups using the ASA scale and also differed in their post-operative condition, in the risk of possible comorbidities development by POSSUM physiological, morbidity, nevertheless were comparable with respect to the risk of possible post-operative death by POSSUM mortality (Table 2). In spite of differences in the severity of comorbidities, no significant post-operative complications were noted in either of the groups. One patient in each group, aged 91 and 94, developed transient post-operative delirium persisting for 2 and 3 days. No mortality was noted in the analyzed material. Farley et al. analyzed, among others, the influence of the comorbidities, abnormalities in laboratory values (high creatinine, glucose, low haemoglobin), opioid use, and hypotensive events in post-operative first day on the length of post-operative hospitalization in 1278 patients who underwent elective THA in an American hospitals in period 2012–2014. The average age of the analyzed patients was 62.3 years (SD 10.7). Length post-operative stay ≥ 3 days in those cases was noted [15].

Lindberg-Larsen et al. analyzed effects of methylprednisolone administered preoperatively on the stability circulatory parameters in the time rehabilitation on the day of surgery THA in the University Orthopedic Hospital in Copenhagen. Although no significant difference was in the circulatory parameters during mobilization at day zero, as many as 40% of the control group subjects interrupted verticalization due to discomfort, as the effect of a higher dose of the opioid preparation, a higher CRP value in the group of patients [22].

The present authors demonstrated that the behaviour of the circulatory system parameters was maintained without any significant differences between the two groups, despite the early rehabilitation (Table 1) on the first post-operative day.

Meta-analyses based on studies employing methylprednisolone and placebo present a significant difference in pain assessment using the VAS scale in first 48 hours post-operatively and opioid agents administered parenterally per 1 kg of body mass [2, 4, 6, 18, 20, 21, 26–28]. Also confirmed by the present study (Figs. 1 and 2) were the differences in the experienced pain as based on the VAS/NRS scale in the M group and the K group measured on day 0 (Fig. 1) and, consequently, the statistical difference between the two groups in the dose of parenterally administered opioid preparations (oxycodone hydrochloride) expressed as mg/kg of body weight given on first 24 hours post-operatively, but in the global dose of other analgesic preparations (metamizole, paracetamol), no significant difference was observed between the two groups (Fig. 2).

Similarly, as in increasingly more numerous reports published last year and currently, the present material confirmed that a single dose of methylprednisolone did not significantly affect local and generalized inflammation. In the literature on the subject, such parameters were evaluated as CRP levels, but also the level of endogenous anti-inflammatory protein pentraxin-3, markers of vascular endothelial dysfunction that initiates inflammation: syndecan-1, thrombomodulin, sE-Selectin or vascular endothelial growth factor (VEGF), interleukin-6, tumor necrosis factor-alfa [16, 17, 29]. Although in 2017 Lindberg-Larsen et al. demonstrated a significantly higher level of pentraxin-3 in the group of patients who received a single dose of methylprednisolone in the first 24 post-operative hours, they documented the absence of local and generalized inflammation development [16]. In another paper, the same group of investigators evaluated the levels of markers of vascular endothelium dysfunction following administration of methylprednisolone vs. placebo after knee arthroplasty. Analyzing the decreasing levels of syndecan-1 and sE-Selectin, thrombomodulin and VEGF, they showed a significantly lower vascular endothelial dysfunction level in the first 24 hours post-operatively in the group of patients administered a single dose of methylprednisolone as compared with the placebo group [17]. Tilinca et al. (2018) analyzed the level of inflammatory markers (interleukin-6, TNF-α) in peri-operative period THA/TKA in the two groups of patients: obese vs. normal body mass index; significant increases in levels of both parameters in the groups of obese subjects [29] were observed. The available literature does not confirm development of local and generalized inflammation [7, 15, 21, 26, 27, 16–18]. Numerous reports point to the fact that the level of CRP does not demonstrate significant differences between the group that received methylprednisolone and the group in which the medication was not employed. In the present material, the analysis focused on such selected inflammatory markers as leukocytosis, and blood and wound drainage fluid CRP levels. The leukocytosis level was significantly higher on day zero in the study group, but the value dropped markedly as early as on days two and three post-operatively (Table 3); in turn, the CRP level was significantly lower in the group “methylprednisolone” as compared with the placebo group. The CRP value in the drainage fluid did not differ between the two groups (Table 3).

As it was demonstrated in the present material, a single dose of methylprednisolone did not significantly affect glycemia fluctuations (Table 3). Similarly, as indicated by data from the literature, the present authors noted in the two groups a transient increase in glycemia, without significant increase in the first 24–48 hours post-operatively [15, 30].

Degenerative changes of peripheral neurons occurring in the process of progressive aging lead to prolonged signal transduction [8, 24, 25]. In consequence, the duration of the effect of peripheral nerve block is prolonged; the intercompartmental peripheral nerve block in the presented material in the group M was almost twice longer contrary to the group K. The material included patients above 65 years of age, whose reaction to local anaesthesia drugs is similar; thus, the difference is from one variable only, i.e., methylprednisolone administration (Fig. 2).

The recently published reports of Kehlet et al. [12, 15, 31, 39, 40] point to this safe aspect of administering a single dose of methylprednisolone that is associated with a quantifiable beneficiary analgesic effect in patients after extensive orthopaedic procedures, with limited post-operative complications [4, 6, 7, 10, 15, 18, 22, 27, 31–33].

In summary, the use of a single dose of methylprednisolone fits in multimodal analgesia in the group of patients above 65 years after surgery unilateral THA: (1) noticeably decreases the level of post-operative pain assessed using the VAS/NRS scales at rest in all time intervals on the day of surgery, and decreases the dose of opioid analgesic medications (oxycodone hydrochloride); (2) no effect has been demonstrated and development of infection symptoms, have noted significantly lower levels of inflammatory markers, CRP levels on all the analyzed post-operative days, leukocytosis level on days two and three post-operatively; (3) no significant difference in glycemia level has been demonstrated; (4) supports the stability of the circulatory system and no adverse effects that would delay patient mobilization and early rehabilitation as per the ERAS protocol.
